# Current state of Alzheimer’s fluid biomarkers

**DOI:** 10.1007/s00401-018-1932-x

**Published:** 2018-11-28

**Authors:** José Luis Molinuevo, Scott Ayton, Richard Batrla, Martin M. Bednar, Tobias Bittner, Jeffrey Cummings, Anne M. Fagan, Harald Hampel, Michelle M. Mielke, Alvydas Mikulskis, Sid O’Bryant, Philip Scheltens, Jeffrey Sevigny, Leslie M. Shaw, Holly D. Soares, Gary Tong, John Q. Trojanowski, Henrik Zetterberg, Kaj Blennow

**Affiliations:** 1 BarcelonaBeta Brain Research Center, Fundació Pasqual Maragall, Universitat Pompeu Fabra, Barcelona, Spain; 2 Unidad de Alzheimer y otros trastornos cognitivos, Hospital Clinic-IDIBAPS, Barcelona, Spain; 3 Melbourne Dementia Research Centre, Florey Institute of Neuroscience and Mental Health, University of Melbourne, Parkville, VIC Australia; 4 Roche Centralised and Point of Care Solutions, Roche Diagnostics International, Rotkreuz, Switzerland; 5 Neuroscience Therapeutic Area Unit, Takeda Development Centre Americas Ltd, Cambridge, MA USA; 6 Genentech, A Member of the Roche Group, Basel, Switzerland; 7 Cleveland Clinic Lou Ruvo Center for Brain Health, Las Vegas, NV USA; 8 Department of Neurology, Washington University in St. Louis, St. Louis, MO USA; 9 AXA Research Fund and Sorbonne University Chair, Paris, France; 10 Sorbonne University, GRC No 21, Alzheimer Precision Medicine (APM), AP-HP, Pitié-Salpêtrière Hospital, Paris, France; 11 Brain and Spine Institute (ICM), INSERM U 1127, CNRS UMR 7225, Paris, France; 12 Department of Neurology, Institute of Memory and Alzheimer’s Disease (IM2A), Pitié-Salpêtrière Hospital, AP-HP, Paris, France; 13 Departments of Epidemiology and Neurology, Mayo Clinic, Rochester, MN USA; 14 Biomarkers, Biogen, Cambridge, MA USA; 15 Department of Pharmacology and Neuroscience; Institute for Healthy Aging, University of North Texas Health Science Center, Fort Worth, TX USA; 16 Department of Neurology and Alzheimer Center, VU University Medical Center, Amsterdam, The Netherlands; 17 Roche Innovation Center Basel, F. Hoffmann-La Roche, Basel, Switzerland; 18 Department of Pathology and Laboratory Medicine, and Center for Neurodegenerative Disease Research, University of Pennsylvania, Philadelphia, PA USA; 19 Clinical Development Neurology, AbbVie, North Chicago, IL USA; 20 Lundbeck, Deerfield, IL USA; 21 Department of Pathology and Laboratory Medicine, Center for Neurodegenerative Disease Research, Perelman School of Medicine at the University of Pennsylvania, Philadelphia, PA USA; 22 Department of Psychiatry and Neurochemistry, The Sahlgrenska Academy at the University of Gothenburg, Mölndal, Sweden; 23 Clinical Neurochemistry Laboratory, Institute of Neuroscience and Physiology, The Sahlgrenska Academy at University of Gothenburg, Mölndal Campus, Sahlgrenska University Hospital, 431 80 Mölndal, Sweden; 24 Department of Molecular Neuroscience, UCL Institute of Neurology, Queen Square, London, UK; 25 UK Dementia Research Institute at UCL, London, UK

**Keywords:** Alzheimer’s disease, Amyloid, Biomarker, Blood, Cerebrospinal fluid, Tau

## Abstract

Alzheimer’s disease (AD) is a progressive neurodegenerative disease with a complex and heterogeneous pathophysiology. The number of people living with AD is predicted to increase; however, there are no disease-modifying therapies currently available and none have been successful in late-stage clinical trials. Fluid biomarkers measured in cerebrospinal fluid (CSF) or blood hold promise for enabling more effective drug development and establishing a more personalized medicine approach for AD diagnosis and treatment. Biomarkers used in drug development programmes should be qualified for a specific context of use (COU). These COUs include, but are not limited to, subject/patient selection, assessment of disease state and/or prognosis, assessment of mechanism of action, dose optimization, drug response monitoring, efficacy maximization, and toxicity/adverse reactions identification and minimization. The core AD CSF biomarkers Aβ42, t-tau, and p-tau are recognized by research guidelines for their diagnostic utility and are being considered for qualification for subject selection in clinical trials. However, there is a need to better understand their potential for other COUs, as well as identify additional fluid biomarkers reflecting other aspects of AD pathophysiology. Several novel fluid biomarkers have been proposed, but their role in AD pathology and their use as AD biomarkers have yet to be validated. In this review, we summarize some of the pathological mechanisms implicated in the sporadic AD and highlight the data for several established and novel fluid biomarkers (including BACE1, TREM2, YKL-40, IP-10, neurogranin, SNAP-25, synaptotagmin, α-synuclein, TDP-43, ferritin, VILIP-1, and NF-L) associated with each mechanism. We discuss the potential COUs for each biomarker.

## Introduction

Worldwide, approximately 50 million people are living with dementia, with Alzheimer’s disease (AD) comprising 60–70% of cases [391]. AD is a progressive, neurodegenerative disease characterized clinically by cognitive decline and behavioural disturbances and pathologically by the accumulation of amyloid beta (Aβ) plaques and neurofibrillary tangles formed by tau fibrils, together with degeneration of neurons and their synapses, glial activation, and neuroinflammation [37, 149, 314]. The incidence of AD increases with age, and the prevalence is growing as a result of the ageing of the population [6]. To date, no disease-modifying therapy (DMT) has been successful [18]. This lack of success may be partly explained by AD having a complex aetiology and considerable heterogeneity in its pathophysiology, and by limitations in past clinical trial designs, which have generally enrolled participants later in the course of the disease (e.g. mild-to-moderate AD), and which did not enrich for Aβ-positive individuals, resulting in substantial misclassification (i.e. inclusion of participants without Aβ pathology) [12, 241, 317].

Biomarkers hold promise for enabling more effective drug development in AD and establishing a more personalized medicine approach [126, 127, 314]; they may soon become essential in staging, tracking, and providing a more quantitative categorization of the disease, as well as for documenting the effect of potential therapeutics. These points are underscored in the 2018 draft guidance documents issued by both the US Food and Drug Administration (FDA) (Early Alzheimer’s disease: developing drugs for treatment; draft guidance for industry) [96] and the European Medicines Agency’s (EMA) Committee for Medicinal Products for Human Use (CHMP) (Guideline on the clinical investigation of medicines for the treatment of Alzheimer’s disease) [78]. Fluid biomarkers have the potential to be easy to implement in clinical trials, and several biomarkers reflecting different pathophysiological mechanisms can be analyzed in the same sample. Furthermore, cerebrospinal fluid (CSF) or blood may provide a window for detection of some biomarkers that cannot be identified via brain imaging [125].

CSF represents a logical source for developing viable biomarkers in AD given its direct interaction with the extracellular space in the brain, thus potentially reflecting the associated pathophysiological alterations [32]. The overall safety record of lumbar puncture is strongly supported by extensive meta-analyses [76, 262]. However, fluid biomarkers are unable to reflect brain regional pathogeographies, which may be particularly important during early AD [47, 281]. Other limitations of CSF include the relative invasiveness of CSF collection by lumbar puncture, limited access and acceptability in some countries, the inability to collect samples from large populations especially if serial measures are needed, concerns over slowing for subject recruitment into clinical trials, educational gaps on the safety of lumbar puncture, development and validation of CSF assays, and clinical utility. Some of the limitations of CSF have prompted research efforts into the development and validation of diagnostic or prognostic AD biomarkers in blood [215, 216]. Indeed, the Biofluid Based Biomarkers Professional Interest Area [of the Alzheimer’s Association International Society to Advance Alzheimer’s Research and Treatment (ISTAART)], an international working group of leading AD scientists, has been established to scrutinize potential blood-based biomarkers and to provide standards for the collection of biofluids [128, 140, 268, 269].

The ideal fluid biomarker for AD would be reliable, reproducible, non-invasive, simple to measure, and inexpensive [360], as well as easy to implement into large populations such as clinical trials and the primary care setting. Importantly, biomarkers used in drug-development programmes should be qualified for a specific context of use (COU); these include (but are not limited to) patient/clinical trial diagnosis and subject selection, assessment of disease state and/or prognosis, assessment of mechanism of action, dose optimization, drug–response monitoring, efficacy maximization, and toxicity/adverse reaction identification and minimization [95]. For successful AD drug development, it is critical to ensure that subjects enrolled into clinical trials are those who have AD pathology and are most likely to progress along the disease continuum. Fluid biomarkers could have an important role in clinical trial subject selection (including subject enrichment or stratification) [123, 126, 127], and could be useful for measuring target engagement of the drug and the impact of the drug on the pathogenic mechanisms [184, 279]. Additionally, fluid biomarkers, especially blood biomarkers, could be used in early screening in primary care to identify potential clinical trial subjects and patients at risk of AD, thereby improving early diagnosis and enabling longitudinal tracking of various disease indicators over extended periods of time [22, 128].

Currently, three core AD CSF biomarkers are included in research guidelines for AD and are being increasingly used in clinical trials as inclusion criteria and/or outcome measures: CSF amyloid beta 42 (Aβ42), total tau (t-tau), and tau phosphorylated at threonine 181 (p-tau) [75, 214, 217, 247, 298]. These biomarkers have been validated as core CSF biomarkers of AD pathophysiology [33, 99, 122, 124]. Qualification opinions have also been published for CSF Aβ42 and t-tau by the EMA, supporting their use as patient-selection tools [153]. Although these core biomarkers are now recognized for their diagnostic utility, there is a need to identify additional fluid biomarkers for other COUs such as subject enrichment, risk stratification, prognosis, and (eventually) drug–response monitoring, and to better understand the complex heterogeneity of AD pathology [78, 96]. Several novel biomarkers have been proposed; some have been extensively investigated, but they have yet to be validated and integrated into guidelines for use in clinical practice and drug development [99, 206].

**Fig. 1:**
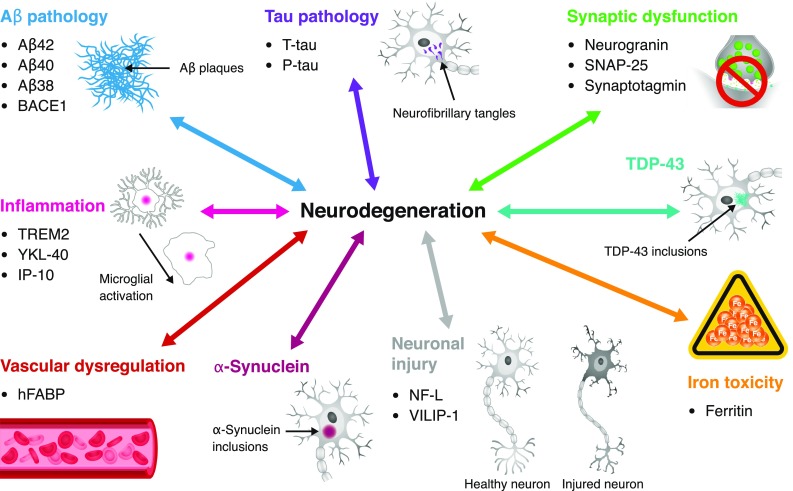
Pathological mechanisms implicated in AD and associated fluid biomarkers. In this figure, the arrows reflect hypothetical relationships, not direct causal links between the pathological mechanisms and neurodegeneration. Only select pathological mechanisms (and associated biomarkers) of AD are represented. *Aβ38* amyloid beta 38, *Aβ40* amyloid beta 40, *Aβ42* amyloid beta 42, *AD* Alzheimer’s disease, *BACE1* β-site amyloid precursor protein cleaving enzyme 1, *hFABP* heart-type fatty acid-binding protein, *IP*-*10* interferon-γ-induced protein 10, *NF*-*L* neurofilament light, *P*-*tau* phosphorylated tau, *SNAP*-*25* synaptosome-associated protein 25, *TDP*-*43* transactive response DNA-binding protein 43, *TREM2* triggering receptor expressed on myeloid cells 2, *T*-*tau* total tau, *VILIP*-*1* visinin-like protein 1

This review summarizes some of the pathological mechanisms implicated in sporadic AD (Fig. 1) and highlights several established and novel fluid biomarkers associated with each mechanism. For each biomarker, a summary of published studies, the stage of assay development (Table 1), and the potential COU (Table 2) are discussed. Most of the fluid biomarkers examined in this review are CSF biomarkers, owing to the limited number of published studies on blood-based biomarkers. It should be noted that unselected biomarker combinations (“panels”) identified through “omics” technologies are not included; this topic has been recently reviewed elsewhere [46, 219, 222, 260].

**Table 1:** Summary of selected candidate AD fluid biomarkers

Biomarker	Stage of clinical validation	Levels in AD vs healthy controls	Stage of assay development	
			CSF	Plasma/serum
Aβ42	CSF Aβ42 is accepted as part of research criteria [75, 247] IWG-2 criteria recommend using CSF Aβ42 in combination with CSF t-tau or p-tau [75] Many studies on plasma but inconsistent results [160, 227, 271, 273, 274, 377]	Consistently decreased in CSF Inconsistent results in plasma, although recent studies have shown a decrease	Commercially available assays, including fully automated (IVDs in Europe)	Commercially available assays
Aβ40	Many studies on CSF and plasma Inconsistent results for Aβ40 alone [73, 83, 160, 243, 248, 271, 338] Consistent results for ratio of CSF Aβ42/Aβ40 [161, 202, 205, 275, 276, 294] Consistent results for ratio of plasma Aβ42/Aβ40 [31, 87, 179, 261, 273, 371, 373, 377, 383]	Aβ40 alone: inconsistent results in CSF and plasma Ratio of Aβ42/Aβ40: consistently decreased in CSF and plasma	Commercially available assays (IVDs in Europe)	Commercially available assays
Aβ38	Several studies on CSF Aβ38 alone Inconsistent results for Aβ38 alone [161, 243, 271] Very few studies on CSF Aβ42/Aβ38 [161, 259] One study on plasma Aβ38 [273]	Aβ38 alone: inconsistent results in CSF and plasma Ratio of Aβ42/Aβ38: decreased in CSF but data are limited	Commercially available assays	Commercially available assays
BACE1	Several studies on CSF Inconsistent results [79, 81, 258, 283, 311, 392, 401, 407] Very few studies on plasma [330, 393]	Inconsistent results in CSF but most studies showed increased levels/activity Increased activity in plasma but data are limited	Commercially available assays	Commercially available assays
T-tau	CSF t-tau is accepted as part of research criteria [75, 247] IWG-2 criteria recommend using CSF t-tau in combination with CSF Aβ42 [75] Several studies on plasma Consistent results [61, 68, 245, 251, 271, 404]	Consistently increased in CSF Consistently increased in plasma	Commercially available assays, including fully automated (IVDs in Europe)	Commercially available assays
P-tau	CSF p-tau is accepted as part of research criteria [75, 247] IWG-2 criteria recommend using CSF p-tau in combination with CSF Aβ42 [75] Few studies on plasma or serum Consistent results [329, 358, 395]	Consistently increased in CSF Increased in plasma and serum but data are limited	Commercially available assays, including fully automated (IVDs in Europe)	In-house assays
hFABP	Several studies on CSF Consistent results [53, 67, 119, 146, 154, 201, 271] Very few studies on plasma or serum [254, 271]	Consistently increased in CSF No change in plasma or serum but data are limited	Commercially available assays	Commercially available assays
TREM2	Few studies on CSF Inconsistent results [38, 107, 139, 141, 287, 344, 346] Few studies on blood [145, 255, 352]	Inconsistent results in CSF but most studies showed an increase No change in plasma levels but data are limited; increased mRNA and protein levels in blood cells but data are limited	Commercially available assays	Commercially available assays
YKL-40	Several studies on CSF Inconsistent results [2–4, 11, 23, 57, 106, 138, 176, 244, 271, 303, 347, 384] Very few studies on plasma [54, 57]	Inconsistent results in CSF but most studies showed an increase Increased in plasma but data are limited	Commercially available assays	Commercially available assays
IP-10	Few studies on CSF Inconsistent results [29, 101, 384] Very few studies on plasma or serum [102, 154]	Inconsistent results in CSF Inconsistent results in plasma or serum	Commercially available assays	Commercially available assays
Neurogranin	Many studies on CSF Inconsistent results [63, 64, 138, 175, 189, 190, 221, 242, 282, 291, 310, 347, 354, 361] Few studies on plasma [63, 110, 190, 389]	Inconsistent results in CSF but most studies showed an increase No change in plasma but studies are limited; decreased in plasma neuronally derived exosomes but data are limited	Commercially available assays	Commercially available assays
SNAP-25	Two studies on CSF [36, 347] No studies on plasma	Increased in CSF but data are limited Unknown for plasma	Commercially available assays	Commercially available assays
Synaptotagmin	One study on CSF [270] One study on plasma [110]	Increased in CSF but data are limited Decreased in plasma neuronally derived exosomes but data are limited	Commercially available assays	Commercially available assays
α-Synuclein	Few studies on CSF Inconsistent results [27, 170, 172, 183, 232, 339, 366] Very few studies on plasma [49, 331]	Inconsistent results in CSF but most studies showed an increase No change in plasma but data are limited	Commercially available assays	Commercially available assays
TDP-43	No studies on CSF Very few studies on plasma [97, 387]	Unknown for CSF Increased in plasma but data are limited	Commercially available assays	Commercially available assays
Ferritin	Very few studies on CSF [13–15] Very few studies on plasma [15, 112]	No change in CSF but data are limited; increased CSF levels are associated with cognitive decline but data are limited No change in plasma but data are limited; plasma levels are associated with PET Aβ but data are limited	Commercially available assays	Commercially available assays
VILIP-1	Several studies on CSF Inconsistent results [17, 176, 194, 230, 257, 271, 347, 355–357] One study on plasma [355]	Inconsistent results in CSF but most studies showed an increase Increased in plasma but data are limited	Commercially available assays	Commercially available assays
NF-L	Several studies on CSF Consistent results [4, 220, 271, 282, 288, 334, 335, 403] Few studies on plasma or serum [240, 385, 408]	Consistently increased in CSF Increased in plasma but data are limited	Commercially available assays (IVDs in Europe)	Commercially available assays

*Aβ38* amyloid beta 38, *Aβ40* amyloid beta 40, *Aβ42* amyloid beta 42, *AD* Alzheimer’s disease, *BACE1* β-site amyloid precursor protein cleaving enzyme 1, *CSF* cerebrospinal fluid, *hFABP* heart-type fatty acid-binding protein, *IP*-*10* interferon-γ-induced protein 10, *IVD* in vitro diagnostic, *IWG*-*2* International Working Group 2, *NF*-*L* neurofilament light, *P*-*tau* phosphorylated tau, *SNAP*-*25* synaptosome-associated protein 25, *TDP*-*43* transactive response DNA-binding protein 43, *TREM2* triggering receptor expressed on myeloid cells 2, *T*-*tau* total tau, *VILIP*-*1* visinin-like protein 1

**Table 2:**
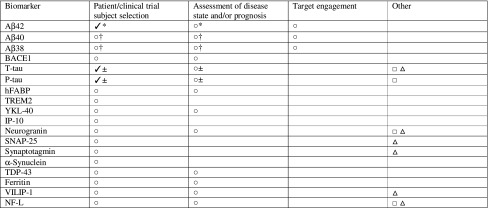
Potential uses for selected candidate AD fluid biomarkers

*Aβ38* amyloid beta 38, *Aβ40* amyloid beta 40, *Aβ42* amyloid beta 42, *AD* Alzheimer’s disease, *BACE1* β-site amyloid precursor protein cleaving enzyme 1, *hFABP* heart-type fatty acid-binding protein, *IP*-*10* interferon-γ-induced protein 10, *NF*-*L* neurofilament light, *P*-*tau* phosphorylated tau, *SNAP*-*25* synaptosome-associated protein 25, *TDP*-*43* transactive response DNA-binding protein 43, *TREM2* triggering receptor expressed on myeloid cells 2, *T*-*tau* total tau, *VILIP*-*1* visinin-like protein 1

^✓^Accepted (validated) use

^○^Potential use, supportive data available

^□^Speculative use for drug response monitoring, no supportive data available

^△^Speculative use for toxicity/adverse reactions minimization, no supportive data available

*Alone or when measured as a ratio with tau

^†^When measured as a ratio with Aβ42

^±^Alone or when measured as a ratio with Aβ42

## Alzheimer’s disease pathological mechanisms

Extracellular plaque deposits of Aβ peptides and intraneuronal tau-containing neurofibrillary tangles (NFTs) and neuropil threads (NTs), are the defining neuropathological features of AD brains [238, 314]. Aβ-plaque deposition is an insidious process that occurs over decades, well before symptoms emerge [156, 379]. Approximately one-third of people over the age of 65 years who are cognitively normal have Aβ-plaque deposition equivalent to that of someone with AD [305]; the significance of this finding is a topic of intensive evaluation.

In AD, comorbid pathology is often present and contributes to the clinical symptoms. For example, there is an increasing burden of cerebrovascular pathology as a function of age, and approximately 30% of AD patients have concomitant cerebrovascular disease [365]. In addition to plaques and tangles, more than half of AD patients also show widespread cortical Lewy bodies (LBs) and Lewy neurites formed by misfolded α-synuclein like those found in patients with Parkinson’s disease dementia (PDD) or dementia with Lewy bodies (DLB) [121, 207]. Conversely, approximately 40% of DLB patients have AD pathology as determined by their CSF-biomarker profile [199]. Furthermore, up to half of AD patients harbour transactive response DNA-binding protein 43 (TDP-43) inclusions that are characteristic of frontotemporal lobar degeneration (FTLD) and sporadic amyotrophic lateral sclerosis (ALS) [7, 50, 159]. Amylin deposits, which are found in the pancreas of most patients with type 2 diabetes mellitus, have also been found in AD (and type 2 diabetes) brains [157]. Thus, although AD is typically characterized by Aβ plaques and NFTs, most AD patients have multiple pathologies and different types of brain proteinopathies [21]. In the Center for Neurodegenerative Disease Research (CNDR) Brain Bank at the University of Pennsylvania, only 35% of 247 autopsy-confirmed AD brains examined for the presence of tau, Aβ, α-synuclein, and TDP-43 deposits had only plaques and tangles as the underlying cause of dementia, while 22% had all four of these pathologies [300]. This finding emphasizes the urgent need for biomarkers that can indicate the presence of multiple pathologies in AD patients, with the methodological attributes of being reliable, accessible, and cost-efficient.

## Aβ pathology

The “amyloid cascade hypothesis”, initially proposed in 1992 [134], but essentially embedded in the reports of the initial discovery of the partial Aβ sequence in cerebral vessels [108, 109] and the whole 4 kDa Aβ peptide in plaques [239], is supported by genetic and biochemical data, and has been the dominant model of AD pathogenesis. The model is based on the gradual deposition of fibrillar Aβ as diffuse plaques, which triggers an inflammatory response, altered ion homeostasis, oxidative stress, and altered kinase/phosphatase activity, leading to the formation of NFTs and to widespread synaptic dysfunction and neuronal loss [320]. Notably, recent experimental evidence suggests that the Aβ plaque environment can accelerate the templated spread of tau pathology [136].

Genetic data strongly implicate Aβ in AD pathogenesis [315, 390]. Whether monomeric or aggregated forms are more relevant to the neurodegenerative process remains unknown. Recent reports indicate that soluble Aβ oligomers may be more toxic than Aβ neuritic plaques [320, 321], suggesting that other forms of Aβ may be more relevant to measure. Phase 3 clinical studies of solanezumab, a monoclonal antibody (mAb) that targets monomeric Aβ, resulted in very modest slowing of clinical decline [72], whereas a Phase 1b study of aducanumab, a mAb that targets soluble and insoluble forms of aggregated Aβ, demonstrated robust plaque reduction and a slowing of clinical decline [42, 323].

### Fluid biomarkers of Aβ metabolism and aggregation

#### Aβ peptides

Aβ is generated as the result of the sequential cleavage of amyloid precursor protein (APP) by β-site amyloid precursor protein cleaving enzyme 1 (BACE1) and γ-secretase [45, 394]. The cleavage position of the γ-secretase in the transmembrane domain of APP is imprecise, resulting in the production of Aβ peptides of variable length [166, 289]. Changes in some of these Aβ species have been associated with AD, as discussed below, but little is known about the changes over time in relation to clinical presentation.

#### Aβ42

The 42-amino acid form of Aβ, Aβ42, is a minor component of Aβ peptides in the CSF [290] and plasma [277], but in AD brains, Aβ42 is the principal Aβ peptide in plaques [113, 155]. Decreases in CSF Aβ42 levels in AD patients were first reported by Motter et al. [256]. Several subsequent studies have consistently shown that CSF levels of Aβ42 correlate inversely with plaque load as observed in autopsies and in vivo with positron emission tomography (PET) [82, 114, 158, 343, 353]. CSF Aβ43 is also reported to decrease in AD, but it has similar diagnostic accuracy to CSF Aβ42 [39, 193] so research has focused on the latter.

CSF Aβ42, together with t-tau and p-tau, are biomarkers accepted as supportive of an AD diagnosis [75, 247] (Table 2), and evidence suggests they may be prognostic of disease progression in both cognitively normal individuals [84, 209] and those with mild cognitive impairment (MCI) [5, 9, 91, 133]. CSF Aβ42 has the potential to discriminate AD from FTLD but shows significant overlap with other non-AD dementias [80].

The development of automated assays to measure CSF Aβ42 will reduce variability among samples and laboratories and make it easier to interpret results and implement this biomarker into routine clinical practice [295]. However, several unresolved issues remain when using CSF Aβ42 in clinical trials. First, there needs to be a better understanding of how to interpret changes in CSF Aβ42 levels in AD in response to DMTs, since this is likely to vary with the mechanism of action of the DMT and with the duration of treatment. It seems logical to measure Aβ42 to help determine target engagement of drugs designed to reduce Aβ pathology [88, 208]; however, some trials have reported changes in CSF Aβ42 but no improvement in clinical endpoints [298]. Furthermore, truncated, post-translationally modified fragments of Aβ (e.g. pyroglutamate Aβ42) may be more prone to pathogenic aggregation [25, 117], and consideration needs to be given to what forms of Aβ42 are being measured. In addition, CSF Aβ42 remains relatively stable over time in patients with AD dementia and may have limited utility for monitoring disease progression in this group [34, 41, 367, 402]. Finally, CSF Aβ42 measures are influenced by pre-analytical factors such as the type of collection tube and number of freeze/thaw cycles [198, 284, 368], so it is essential to develop harmonized standard operating procedures for sample collection and handling, as established for biomarker studies in the Alzheimer’s Disease Neuroimaging Initiative (ADNI) [326, 327].

There has been great interest in developing new techniques to measure Aβ42 in blood. Although most studies have failed to show an association between plasma Aβ42 alone and risk of AD or association with PET Aβ [227, 271], recent studies using ultrasensitive analytical assays as well as fully automated immunoassays suggest that plasma Aβ could be a useful screening biomarker. Using an ultrasensitive immunoassay technique (Simoa platform), levels of Aβ42 and the ratio of Aβ42/Aβ40 in plasma were shown to correlate with CSF levels and with Aβ deposition measured by PET [160, 377], and plasma Aβ42/Aβ40 associated with risk of progression to MCI or dementia in cognitively normal individuals with subjective cognitive decline [377]. Ovod et al. used mass spectrometry to demonstrate lower levels of plasma Aβ42 and Aβ42/Aβ40 in subjects with an Aβ-positive PET [273]. In addition to the use of novel technologies, plasma samples could be chemically treated to reduce degradation of Aβ and improve the accuracy of plasma Aβ measurements [278]. Recently, a fully automated immunoassay has been shown to detect plasma Aβ42 and Aβ40 and accurately predict Aβ-positivity (using the CSF Aβ42/40 ratio as reference standard) in cognitively normal, subjective cognitive decline, MCI and AD dementia patients [274].

In summary, CSF Aβ42 is widely accepted and used as an AD biomarker, and both CSF and plasma Aβ42 continue to be intensively studied (Tables 1 and 2). CSF Aβ42 is recognized as a core biomarker for AD diagnosis and is currently being considered by the FDA for qualification for subject selection in clinical trials (Table 2). It shows great promise as a biomarker for prognosis and has the potential to be used during drug trials to help assess target engagement. Plasma Aβ42 may prove to be useful for subject/patient selection (screening) and research is ongoing. There are several commercially available assays, and for CSF Aβ42, there are in vitro diagnostic (IVD) assays in Europe and fully automated assays.

#### Aβ40

Aβ40 is the predominant form of Aβ peptide in the brain [322, 348, 349], CSF [290, 322], and plasma [277], but it does not appear to be as pathogenic as Aβ42 [246]. Aβ40 may have protective effects against Aβ plaque formation [180] but it is the relative amount of Aβ40 to Aβ42 that may be more important than the absolute amounts of either peptide [188].

Research on CSF Aβ40 and its correlation with AD dementia has shown inconsistent results [73, 248, 338], and a meta-analysis by Olsson et al. (data from 25 AD cohorts and 24 control cohorts) found only a minor association [271]. One study demonstrated an association between PET Aβ and CSF Aβ40 levels (as well as CSF Aβ38 and a combination of Aβ40 and Aβ38), although the association was stronger in individuals who were not carriers of the apolipoprotein-E (*APOE*) ε4 allele (a major genetic risk factor for AD) compared with *APOE* ε4-positive individuals [243]. CSF Aβ40 may be useful (together with other biomarkers) in assessing target engagement of drugs such as BACE1 inhibitors, which selectively decrease Aβ40 and Aβ42 [173].

Although CSF Aβ40 shows no consistent change in AD across studies, the ratio of CSF Aβ42/Aβ40 has been shown to be a better predictor of Aβ-positive PET than CSF Aβ42 alone [161, 202, 205, 276], and comparable to the ratios of t-tau/Aβ42 and p-tau/Aβ42 [275, 294]. The Aβ42/Aβ40 ratio also appears to be better than CSF Aβ42 alone at distinguishing AD from non-AD dementias [73, 161]. Assessment of the CSF Aβ42/Aβ40 ratio (together with CSF tau levels), instead of absolute levels of Aβ42, may reduce misdiagnosis of cognitively normal individuals who are low Aβ producers and AD patients who are high Aβ producers [388], and to correct for inter-individual differences in CSF dynamics. The use of the Aβ42/Aβ40 ratio can also help to reduce the impact of pre-analytical factors affecting Aβ42 (and Aβ40) levels [104].

Studies on plasma Aβ40 have had mixed results [160, 248], but the meta-analysis by Olsson et al. (data from 21 AD cohorts and 19 control cohorts) found no difference between AD and controls [271]. In a separate study, plasma Aβ40 did not correlate with Aβ-positive PET in cognitively normal elderly individuals [83]. However, as with CSF, the ratio of Aβ42/Aβ40 in plasma may be more useful than Aβ40 alone; plasma Aβ42/Aβ40 appears to be associated with an increased risk of progression to AD dementia [373, 377], and has shown promise in detecting Aβ-positivity [87, 261, 273, 274, 371, 377] and supporting the diagnosis of AD [31, 179, 383].

Overall, the data suggest that CSF or plasma Aβ40 alone has limited utility as a biomarker for AD diagnosis but could be used to confirm target engagement of certain drugs (Tables 1 and 2). The ratio of Aβ42/Aβ40 in CSF and plasma appears to be useful for subject/patient selection and may be superior to Aβ42 alone (Table 2). Additionally, fluid-based Aβ42/Aβ40 may be useful for prognosis but the data are limited. Commercial assays are available for fluid-based Aβ40 (and Aβ42), and IVD assays are available for CSF Aβ40 (and Aβ42) in Europe. Ongoing research may lead to the availability of validated assays for blood-based Aβ peptides in the future.

#### Shorter Aβ peptides

Aβ peptides shorter than 40 residues have been evaluated for potential utility as AD biomarkers. CSF Aβ38 was included in the meta-analysis by Olsson et al. (eight studies were analyzed) but there was no difference between AD patients and control subjects [271]. However, CSF Aβ38 has been found to correlate with PET Aβ [243] and the ratio of CSF Aβ42/Aβ38 is better at predicting Aβ-positive PET than CSF Aβ42 alone (and comparable to CSF Aβ42/Aβ40) [161]. Furthermore, CSF Aβ42/Aβ38 may be useful for differentiating between AD and DLB [259] and other non-AD dementias [161].

Another possible use for shorter Aβ peptides is to demonstrate target engagement of drugs designed to affect Aβ processing. For example, treatment with γ-secretase modulators is associated with a selective decrease in CSF Aβ42 and Aβ40 and an increase in Aβ38 and Aβ37 [272], so these biomarkers can be used to monitor patients receiving these drugs [341].

In summary, the evidence is limited for fluid-based Aβ peptides < 40 residues (Table 1) but commercial assays are available for CSF Aβ38 and this biomarker has the potential to be used for subject/patient selection (in combination with Aβ42) and to help demonstrate target engagement of γ-secretase modulators (Table 2).

#### Aβ oligomers

Aβ oligomers may play a key role in AD pathogenesis so the accurate detection and quantification of these species in CSF or blood could prove useful. Different technologies have been investigated and some have shown promise; examples include ELISA-based methods in CSF [143, 312, 396] and in plasma [381], single-molecule fluorescence microscopy in CSF [144], and a protein misfolding cyclic amplification assay method in CSF [308]. However, the overall findings have been inconsistent or unsatisfactory (reviewed by Schuster and Funke [318]). A number of methodological issues complicate measurement of Aβ oligomers, including the fact that the oligomeric state of these proteins varies and is affected by numerous factors.

#### BACE1

BACE1 has been shown to have several physiological functions in addition to APP processing [376, 394]. It is believed to be a major protease for cell surface proteolysis, contributing to ~ 19% of identified shed proteins [186], including neuregulin, which has important functions in myelination [94]. Therefore, monitoring of BACE1 activity may be helpful in subjects receiving investigational BACE1 inhibitors.

CSF BACE1 activity and/or protein levels have been reported to be higher in subjects with MCI compared with AD patients or controls [407], and higher in AD patients versus controls [79, 258, 401]. Furthermore, the *APOE* ε4 allele has been associated with increased CSF BACE1 activity in both AD and MCI subjects [81]. CSF BACE1 activity has also been shown to be higher in subjects with MCI who progressed to AD compared with those with stable MCI [401]. However, some studies have found no differences in BACE1 activity among AD, MCI, and control groups [283, 311], and one study found a decline in age-adjusted CSF BACE1 activity in AD patients compared with controls [392]. A recent study of elderly healthy subjects, who received chronic treatment with a BACE1 inhibitor, reported no change in CSF BACE1 levels after BACE1 inhibition, but did find strong correlations between levels of CSF BACE1 and its downstream markers including CSF Aβ42 [362].

Plasma BACE1 has also been studied and has been shown to differentiate AD patients from controls [330, 393]. In addition, plasma BACE1 activity was found to be higher in subjects with MCI who progressed to AD compared with those with stable MCI or AD [330].

Overall, studies of BACE1 have given mixed results, and the association between BACE1 and AD remains unclear (Table 1). Recent research on BACE1 activity in plasma shows potential for subject/patient selection and prognosis (Table 2) but further studies are needed to validate the initial findings. Commercial assays are available to measure both BACE1 protein levels and BACE1 activity.

## Tau pathology

Tau is a microtubule-associated protein comprised of six human isoforms predominantly located in the axon of neurons [177]. Neuronal and/or glial inclusions of tau can be detected in several neurodegenerative diseases, or “tauopathies”, including AD [152], which may be characterized, to some extent, by their tau isoform profile [252]. The NFTs characteristic of AD are composed primarily of hyperphosphorylated tau [19, 196].

The abnormal phosphorylation of tau in AD has been hypothesized to be driven by Aβ pathology [19, 177], although transgenic mice genetically engineered to develop Aβ plaques do not develop tau tangles [197], except after intracerebral injections of AD brain-derived tau [136].

Hyperphosphorylation of tau has several pathogenic effects. It reduces tau’s affinity for microtubules, and increases its likelihood to aggregate and fibrillize [309]. This leads to destabilization of microtubules with subsequent axonal transport failure and neurodegeneration, which can be offset or corrected by microtubule-stabilizing drugs [24, 40, 405]. Hyperphosphorylation of tau is thought to cause its mislocalization to somatodendritic compartments, where it interacts with Aβ to cause synaptotoxicity through the excessive activation of the *N*-methyl-D-aspartate (NMDA) receptors [177]. In addition, hyperphosphorylation of tau is implicated in Aβ-induced cell death [200], possibly via a toxic gain of function mechanism [89].

Studies have shown that the density of neocortical NFTs has a stronger correlation than Aβ plaques with ante-mortem cognitive status (reviewed by Nelson et al.) [263]. This finding, together with the involvement of tau in neurodegenerative processes, has led to increasing interest in tau as a therapeutic target for AD, with several compounds now in the early stages of clinical development [19, 60].

### Fluid biomarkers of tau pathology

#### T-tau and p-tau

CSF t-tau and p-tau (tau phosphorylated at threonine 181), together with CSF Aβ42, are considered core biomarkers to support AD diagnosis [75, 247] (Table 2). Both CSF t-tau and p-tau differentiate AD from controls, and given that CSF p-tau levels are normal in most other dementias, this biomarker is also important for differential diagnosis [33]. In Creutzfeldt–Jakob disease (CJD), CSF t-tau levels are very high (around 20 times higher than in AD), whereas p-tau is close to normal [297, 336]. As with CSF Aβ42, CSF tau has the potential to predict disease progression in cognitively unimpaired individuals [301] and in those with MCI [91, 285]. CSF t-tau has been shown to predict more aggressive disease progression in patients with MCI due to AD or in mild-to-moderate AD [65].

Although CSF t-tau and p-tau are well-established AD biomarkers, their utility for diagnosis of AD is markedly improved when measured in combination with Aβ42 [75]. Hulstaert et al. found that the combination of CSF tau and Aβ42 was better than the individual biomarkers at discriminating AD patients from controls or subjects with other neurological disorders [148]. In the initial CSF study of the ADNI cohort, a logistic regression model combining Aβ42, t-tau, and the *APOE* ε4 allele count showed a stronger association with mild AD than Aβ42, t-tau, p-tau, or tau/Aβ42 alone [326]. Both CSF t-tau/Aβ42 and p-tau/Aβ42 ratios have been shown to outperform any of the individual biomarkers for distinguishing individuals with an Aβ-positive PET [85]. In a study of the Oxford Project to Investigate Memory and Ageing (OPTIMA) cohort, CSF t-tau/Aβ40 and p-tau/Aβ42 were the best discriminators of autopsy-confirmed AD from controls [319]. The combination of CSF tau and Aβ42, in particular, p-tau/Aβ42, has also shown promise for differentiating AD from other dementias [299, 319].

The combination of tau and Aβ markers has also demonstrated their utility for predicting disease progression. CSF t-tau/Aβ42 and p-tau/Aβ42 have been shown to predict cognitive decline in cognitively normal individuals [84], and the combination or ratios of tau (t-tau or p-tau) and Aβ42 have been shown to be better at predicting progression from MCI to AD than the individual biomarkers [91, 374]. Furthermore, the EMA approved CSF Aβ42/t-tau for use as an enrichment biomarker in a study of a γ-secretase inhibitor [77]. More recently, the synergistic interaction between CSF p-tau and Aβ imaging was found to be associated with the progression from MCI to AD dementia [280].

CSF t-tau and p-tau are frequently measured in clinical trials but, as with CSF Aβ42, the relationship between clinical endpoints or therapeutic drug (interventional) response and these biomarkers is unclear [298], owing to their small longitudinal variation [34, 41] and the lack of DMTs that precludes testing their performance. Other challenges include variability in measured values due to pre-analytical and analytical factors, and the lack of consensus on cut-off values [98]. However, it is feasible that CSF tau could be used to assess target engagement of tau-targeted drugs [174, 363].

In addition to the CSF tau biomarkers, plasma tau has also been evaluated and has shown potential for clinical utility. The meta-analysis by Olsson et al. (data from six AD cohorts and five control cohorts) found an association between plasma t-tau and AD [271]; however, no significant difference in plasma t-tau has been reported between MCI subjects and controls [61, 245, 404]. Elevated plasma t-tau is associated with lower grey matter density but the brain atrophy pattern associated with plasma t-tau is different from that of CSF t-tau [68]. Longitudinally, higher levels of plasma t-tau have been associated with greater cognitive decline and risk of MCI [245, 251]. Notably, the relationship between plasma t-tau and cognition was independent of elevated brain Aβ [251]. These findings suggest that plasma t-tau could be useful as a screening tool or a prognostic marker for non-specific cognitive decline in cases where acute central nervous system (CNS) injury has been ruled out. Blood-based p-tau has also been measured in a few recent studies and found to be elevated in AD patients [329, 358, 395] and MCI subjects [329, 395] compared with controls. In addition, platelet-derived tau has been explored, and preliminary studies suggest that the ratio of high molecular weight to low molecular weight tau is higher in AD than in controls [264, 337]. However, the role of platelet-derived tau is not clear; it could either be a confounder or help to clarify the relationship between central and peripheral compartment tau measurements.

Although most tau biomarker research has focused on t-tau and p-tau, studies suggest that a variety of other tau peptides and fragments can be detected in CSF [250] and in serum [150], and some may have potential as AD biomarkers. For example, one study found that 11 (of 47) different tau phosphopeptides were upregulated in AD patients relative to controls [306], while another study found that non-phosphorylated tau also has potential as a diagnostic biomarker [204]. These initial studies may lead to further research into novel tau biomarkers, which may be especially helpful in the development of tau-directed therapies.

In summary, CSF t-tau and p-tau are widely accepted and used in AD research (Tables 1 and 2). They are recognized as core biomarkers to support the diagnosis of AD and are currently being considered for qualification by the FDA for subject selection in clinical trials (Table 2). CSF tau also shows promise as a biomarker for prognosis and target engagement. Commercial assays are available for both CSF t-tau and p-tau, including IVD assays in Europe and fully automated assays. There has been recent renewed interest in plasma t-tau, which shows potential for subject/patient selection (screening) and for prognosis (Table 2). In addition, research into other tau biomarkers (blood-based p-tau, platelet-derived tau, tau peptides/fragments) is ongoing but is still in its early stages.

## Vascular dysregulation

Concurrent cerebrovascular disease is more common in AD than in most other neurodegenerative disorders [365] and vascular dysregulation as a contributing factor to AD has been a long-standing hypothesis for AD pathogenesis [296, 340]. The time sequence of the impact of vascular dysregulation has been debated, but recent work supports the possibility that these changes may be an early pathological event that precedes Aβ pathology. The spatiotemporal changes in various neuroimaging, plasma, and CSF biomarkers from the ADNI cohort, suggest that vascular dysregulation is the earliest and strongest pathological factor associated with late-onset AD, followed by Aβ deposition, glucose metabolism dysregulation, functional impairment, and grey matter atrophy [154].

Vascular dysregulation reduces oxygen and nutrient supply to the brain, causing cell damage and dysfunction of the blood–brain barrier, which lead in turn to neurotoxic effects such as oxidative stress and inflammation [69]. The hypoxic conditions are thought to increase the accumulation of Aβ peptides through the activation of BACE1 and γ-secretase [307]. Additionally, disruption of the blood–brain barrier has been suggested to impair clearance of Aβ peptides from the brain [48].

### Fluid biomarkers associated with the vascular system

#### hFABP

Heart-type fatty acid-binding protein (hFABP), which has been proposed as a biomarker of myocardial infarction [1], was the CSF analyte with the highest degree of abnormalities in the spatiotemporal analysis of the ADNI cohort [154]. It was also identified as a potential AD biomarker in an independent cohort [146]. FABP showed associations with CSF Aβ42 levels but not with cognitive impairment [201]. In the meta-analysis by Olsson et al., CSF hFABP had a moderate association with AD (data from five AD and control cohorts), with a lower degree of change in AD versus controls than seen for t-tau [271]. CSF hFABP has also been shown to predict progression from MCI to AD [119], correlate with brain atrophy among individuals with low CSF Aβ42 [67], differentiate AD and DLB from Parkinson’s disease (PD) and other neurological diseases [53], and correlate with cognitive impairment in a cohort of patients with different neurodegenerative diseases [53]. The source of hFABP in CSF is uncertain but it is highly expressed in the brain where hFABP levels are second only to levels in muscle tissues (https://www.proteinatlas.org/ENSG00000121769-FABP3/tissue).

Serum hFABP was included in the meta-analysis by Olsson et al. and showed no association with AD (data from two AD and control cohorts) [271]; one study suggested it may be useful for differentiating between AD and DLB when measured as a ratio with CSF tau [254].

In summary, recent data for hFABP suggest that it may play a more important role in AD than previously thought (Table 1). CSF hFABP could be useful for both subject/patient selection and prognosis (Table 2) but further studies are needed to confirm these hypotheses. Commercial assays are available for CSF and serum hFABP.

## Inflammation/glial activation

Inflammation has been proposed as a contributor to AD pathogenesis [37, 44]. Aβ plaques and NFTs induce an immune response in the brain, which is characterized by activated glial cells [37]. Microglia and astrocytes are the two main types of glial cells implicated in the pathogenesis of AD [37]. Microglia, the resident immune effector cells of the CNS, are important for brain homeostasis as well as immune responses [52]. Astrocytes are the most abundant type of glial cell in the CNS. They have important roles in homeostasis, synaptogenesis, signal transmission, and synaptic plasticity, and provide trophic and metabolic support to neurons [342].

The activation of glial cells serves to protect the brain; however, uncontrolled and prolonged activation can lead to detrimental effects that override the beneficial effects [37]. In this condition, glial cells lose some of their homeostatic functions and acquire a pro-inflammatory phenotype. The release of pro-inflammatory molecules, reactive oxygen species, and nitric oxide contribute to neuronal cell death. In addition, pro-inflammatory molecules increase Aβ synthesis as well as tau hyperphosphorylation [37].

### Fluid biomarkers of inflammation/glial activation

#### TREM2

Triggering receptor expressed on myeloid cells 2 (TREM2) is expressed by many cells of the myeloid lineage, including microglial cells in the CNS, and has several physiological functions including the regulation of myeloid cell number, phagocytosis, and inflammation [162]. TREM2 expression is upregulated in AD brains, where it may have a protective effect in the early stages, through the phagocytic clearance of Aβ, but a pathogenic effect in the later stages, through activation of the inflammatory response [162]. Rare *TREM2* gene variants have been associated with an increased risk of developing AD [59, 116, 304, 332]. *TREM2* haplodeficiency in mice and humans has been associated with increased axonal dystrophy and p-tau accumulation around Aβ plaques [400].

A soluble variant, sTREM2, can be detected in CSF and has the potential to be used as a biomarker for AD. One study found that CSF sTREM2 levels were increased in autosomal dominant AD mutation carriers 5 years before expected symptom onset but after initial Aβ deposition (as measured by PET) and changes in CSF Aβ42 and t-tau [344]. Some studies have found slightly higher CSF sTREM2 levels in AD [38, 141, 287, 346] and MCI groups [38] compared with controls, and in subjects with MCI due to AD compared with other AD groups (preclinical AD or AD dementia) [346]. However, one study found no difference between patients with AD or MCI and cognitively normal controls [139]. In patients with MCI, elevated CSF sTREM2 levels correlated with increased grey matter volume and reduced diffusivity, suggesting a role for TREM2 in the regulation of the neuroinflammatory response in early AD [107].

Levels of TREM2 mRNA in peripheral blood mononuclear cells and TREM2 protein expression on monocytes have been reported to be higher in patients with AD than in controls, and inversely correlated with cognitive performance [145]. In the same study, there was also a trend for upregulation of TREM2 protein on granulocytes and in plasma but this was not statistically significant [145]. Subsequent studies by other groups also found increased peripheral TREM2 mRNA expression in AD compared with controls [255, 352].

In summary, a few studies have observed increased levels of CSF sTREM2 and peripheral TREM2 expression in AD (Table 1), suggesting possible use in subject/patient selection (Table 2) but additional research is required to validate these findings. Commercial assays are available for the measurement of TREM2 protein.

#### YKL-40

YKL-40 (or chitinase-3-like protein 1) is upregulated in a variety of inflammatory conditions and cancers, and may have a role in promoting inflammation and angiogenesis [211]. In AD, YKL-40 is expressed in astrocytes near Aβ plaques [57] and correlates positively with tau pathology [293], suggesting a role for YKL-40 in the inflammatory response in AD and other tauopathies.

Several studies have shown that CSF YKL-40 levels are higher in AD patients compared with controls [4, 11, 23, 57, 176, 303, 384], and in the late preclinical AD stages compared with early preclinical stages [2]. The meta-analysis by Olsson et al. found that the degree of increase is modest (data from six AD cohorts and five control cohorts) compared with the change in neuronal proteins such as t-tau and neurofilament light (NF-L) [271]. However, a recent study of the ADNI cohort found no significant difference between the AD and cognitively normal groups, although levels were higher in AD versus MCI Aβ-negative (based on CSF Aβ42) subjects [347]. Longitudinally, all groups showed an increase in CSF YKL-40 over time, but the change was statistically significant only in the MCI Aβ-positive group (mean follow-up was 4 years) [347]. CSF YKL-40 levels have been shown to correlate with neuroimaging parameters, including cortical thickness in AD-vulnerable areas in subjects who were Aβ42-positive (by CSF) [3] and grey matter volume in *APOE* ε4 carriers [106].

Higher levels of CSF YKL-40 and YKL-40/Aβ42 ratio have been associated with increased risk of progression from normal cognition to MCI [57]. Levels of CSF YKL-40 have been found to predict progression from MCI to AD and increase longitudinally in MCI and AD patients but not in cognitively normal individuals [176]. CSF YKL-40 has also been shown to differentiate AD from DLB, PD [384], FTLD [23], and non-AD MCI [138], although one early study found no differences among diagnostic groups [244].

Plasma YKL-40 has also been assessed as an AD biomarker, and elevated levels have been reported in patients with mild AD [57] and early AD [54] compared with controls. However, plasma YKL-40 did not demonstrate utility for predicting cognitive decline [57].

In summary, the available evidence supports a role for CSF YKL-40 as a biomarker of neuroinflammation or astrogliosis in AD and other neurodegenerative diseases (reviewed by Baldacci et al. [20]), with the potential to aid subject/patient selection and prognosis (Tables 1 and 2). Plasma YKL-40 could also be useful for subject/patient selection, but further studies are needed. Commercial assays are available.

#### Other inflammatory markers

Interferon-γ-induced protein 10 (IP-10), which has roles in angiogenesis as well as inflammation and is secreted by a variety of cells [10, 223], has been reported to be increased in the CSF of patients with MCI and mild AD but not in severe AD [101]. However, in another study, IP-10 levels were not increased in the AD group [384]. In a recent study in asymptomatic older adults, increased levels of CSF IP-10 were associated with increased levels of CSF t-tau and p-tau [29].

IP-10 was the plasma analyte with the highest degree of abnormalities in a spatiotemporal analysis of biomarkers from the ADNI cohort [154]. However, a previous study found no association between serum IP-10 and AD [102].

Overall, very few studies have investigated IP-10 in AD and the results have been mixed (Table 1). Potentially, CSF or blood-based IP-10 could support subject/patient selection (Table 2), but further research is warranted to clarify the role of IP-10 in AD. Commercial assays are available.

Many other inflammatory markers have been investigated for their potential use as biomarkers for AD, but results have been inconsistent [142, 350]. In a meta-analysis of 40 studies on blood and 14 on CSF, AD patients had higher levels of interleukin (IL)-6, tumour necrosis factor (TNF)-α, IL-1β, transforming growth factor-β (TGF-β), IL-12, and IL-18 in blood, and higher levels of TGF-β in CSF, compared with controls [350]. In a more recent meta-analysis of 175 studies on blood, increased IL-1β, IL-2, IL-6, IL-18, interferon-γ, homocysteine, high-sensitivity C reactive protein, C-X-C motif chemokine-10, epidermal growth factor, vascular cell adhesion molecule-1, TNF-α converting enzyme, soluble TNF receptors 1 and 2, α1-antichymotrypsin and decreased IL-1 receptor antagonist and leptin were found in patients with AD compared with controls [191]. These findings strengthen the evidence that AD is accompanied by inflammatory responses, although the effects of age and sex and the precise roles of different inflammatory mediators are still to be established. A more systematic, within- and between-subject, rigorous longitudinal evaluation may improve the utility of inflammatory markers in AD and other neurodegenerative diseases.

## Synaptic dysfunction

Synaptic dysfunction and synapse loss are early events in AD pathogenesis [167, 218, 359]. Notably, hippocampal synapse loss and impaired synaptic function were detected in 3-month-old tau transgenic mice, when pathological tau was detectable biochemically but before microscopically visible neurofibrillary tau tangles emerged [397]. The level of synaptic loss in post-mortem brains has been found to correlate with pre-mortem cognitive function in individuals with MCI or early AD [62, 313]. The synaptic pathology in AD is found throughout the neuropil, without any clear accentuation in relation to plaques [30, 235]. Importantly, the synaptic loss in AD is more severe than the neuronal loss in the same cortical region [137, 237]. A PET tracer has recently been developed that binds to synaptic vesicle glycoprotein 2A (SV2A) and can be used to quantify synaptic density in vivo; this could be used to complement existing AD imaging tools in the future [93].

Evidence suggests that NMDA receptors are central to the synaptic dysfunction observed in AD. Overstimulation of NMDA receptors triggers an excessive influx of calcium, which in turn can lead to a series of downstream events that culminate in synaptic dysfunction and apoptosis [167, 369]. Aβ oligomers are thought to contribute to NMDA activation, possibly by causing an aberrant rise in extrasynaptic glutamate levels [369].

### Fluid biomarkers of synaptic dysfunction

#### Neurogranin

Neurogranin is predominantly expressed in dendritic spines and is involved in post-synaptic signalling pathways through the regulation of the calcium-binding protein calmodulin [70]. Animal models and genetic studies have linked neurogranin to cognitive function and synaptic plasticity [70]. Notably, CSF neurogranin has been proposed as a marker of synaptic degeneration [361] and, together with other synaptic proteins, holds promise to serve as a novel candidate marker for AD [218].

CSF neurogranin levels are higher in AD [63, 175, 190, 221, 242, 291, 310, 347, 354, 361] or MCI patients [291, 347] compared with controls or non-AD dementia patients [354]. Higher levels of CSF neurogranin have been reported in AD compared with MCI [138, 291], although there was no significant difference between AD and MCI Aβ-positive (based on CSF Aβ42) groups in a recent study of the ADNI cohort [347]. Also in the ADNI study, CSF neurogranin levels decreased longitudinally in the AD group (mean follow-up was 4 years) but there was no significant longitudinal change in any other group [347]. Neurogranin is processed to a series of C-terminal peptides before release into the CSF [190], but the relevance of the individual peptides is unknown. However, one study that used an assay specific for C-terminally truncated neurogranin observed increased levels in MCI patients but no significant difference between AD patients and controls [64]. CSF neurogranin has been shown to predict disease progression in several studies [175, 189, 291, 354] including future cognitive impairment in cognitively normal controls [354]. In addition, CSF neurogranin levels have been found to correlate with brain atrophy but only in individuals with Aβ pathology [282].

To date, no significant differences have been reported in plasma levels of neurogranin between patients with AD and controls [63, 190]. However, levels of neurogranin in neuronally derived exosomes in plasma have been found to be lower in AD patients compared with controls [110, 389], as well as in MCI subjects who progressed to AD compared with stable MCI subjects [389].

Overall, the available data indicate that CSF neurogranin (and potentially, plasma neuronally derived exosomes) could be useful as an AD biomarker for subject/patient selection and prognosis (Tables 1 and 2), although results may vary depending on the neurogranin fragment being measured (full-length vs C-terminal peptides and C-terminus intact vs truncated). Commercial assays are available.

#### SNAP-25 and synaptotagmin

The exocytosis of synaptic vesicles for neurotransmitter release is a complex process, mediated by several proteins including the SNARE (soluble *N*-ethylmaleimide-sensitive factor attachment protein receptor) complex and the calcium sensor protein synaptotagmin [131]. Post-mortem studies on AD brains have shown altered levels of several synaptic proteins, including synaptosome-associated protein 25 (SNAP-25), a component of the SNARE complex [74], and synaptotagmin [236, 351].

CSF levels of SNAP-25 [36, 347] and synaptotagmin [270] have been assessed and found to be elevated in patients with AD or MCI compared with controls. In a study of the ADNI cohort, baseline CSF SNAP-25 levels were higher in AD and MCI Aβ-positive (based on CSF Aβ42) groups than the cognitively normal (Aβ-positive or -negative) and MCI Aβ-negative groups [347]. CSF SNAP-25 levels decreased longitudinally in the AD group (mean follow-up was 4 years) but there was no significant longitudinal change in any other group [347]. No studies have been published to date on blood-based SNAP-25, but synaptotagmin levels in plasma neuronally derived exosomes have been reported to be lower in AD patients than in controls [110]. The data are limited but suggest there could be a role for the synaptic proteins, SNAP-25 and synaptotagmin, as AD biomarkers for subject/patient selection (Tables 1 and 2). Commercial assays are available for both SNAP-25 and synaptotagmin.

## α-Synuclein pathology

α-Synuclein is an abundant neuronal protein, predominantly localized in the presynaptic terminals, and involved in vesicle fusion and neurotransmitter release [181]. Aggregates of α-synuclein are the main component of LBs, which are intracellular inclusions characteristic of certain neurodegenerative diseases termed α-synucleinopathies [181]. Primary α-synucleinopathies include PD, PDD, DLB, and multiple system atrophy [181]; however, α-synuclein aggregates are also found in approximately half of sporadic AD brains [121] and Down’s syndrome brains with concomitant AD pathology [213], and in almost all cases of familial AD with *PSEN 1* mutations [203].

α-Synuclein oligomers have been shown to have multiple toxic effects including inflammation, synaptic dysfunction, compromised cell membrane integrity, and impaired intracellular protein degradation [151, 406]. Furthermore, there is growing evidence that α-synuclein may act in a prion-like manner such that misfolded α-synuclein can be propagated from cell to cell [35, 118, 164, 370], even in wild-type non-transgenic mice [229]. The relationship between AD pathology and α-synuclein is unclear, although studies suggest that α-synuclein can act synergistically with both tau [105] and Aβ [234] to promote their aggregation and accumulation.

### Fluid biomarkers of α-synuclein pathology

#### α-Synuclein

Although CSF α-synuclein was developed as a candidate biomarker for PD, levels of CSF α-synuclein have been found to be higher in patients with MCI [183] and AD [183, 232, 339] compared with controls. However, in one study, no differences were reported between diagnostic groups except for higher levels in rapid progressors (MCI patients who developed AD during the 2-year study and had a short duration of symptoms before the study) [27]. CSF α-synuclein shows a strong correlation with CSF t-tau and a weaker correlation with p-tau in AD, but a subset of patients in the ADNI cohort had a mismatch—high p-tau accompanied by low α-synuclein levels—it was hypothesized that this CSF signature could represent concomitant LB pathology in AD patients [366].

CSF α-synuclein has been assessed as a biomarker in PD and other neurodegenerative diseases [66, 171, 253] and is a major focus area (together with tau and Aβ) of the Parkinson’s Progression Marker Initiative (PPMI) [168, 169]. α-Synuclein in plasma [212], and even in salivary secretions [380], has been investigated in PD.

CSF α-synuclein levels have been reported to be slightly lower in PD compared with AD [253] or controls [66, 168, 169, 253]. CSF α-synuclein levels were lower in DLB patients compared with AD patients in some studies [172, 253, 339], most often with a large overlap between the diagnostic groups, but the opposite was observed in one study [170]. Importantly, CSF α-synuclein levels are many-fold higher in CJD than in PD [171, 253]. Commercial assays are available for total α-synuclein and one has been clinically validated for the diagnosis of sporadic CJD [225].

Most currently available assays for α-synuclein have been designed to measure total amounts of the protein and not LB-specific fragments, although phosphorylated α-synuclein has been detected in CSF of PD patients [382]. There are reports of increased CSF concentrations of α-synuclein oligomers in CSF of PD patients [132, 233, 364], and recent publications on sensitive assays that appear to detect the minute amounts of putative seeds of α-synuclein oligomers in CSF [86, 325].

Plasma levels of α-synuclein have been reported to be elevated in patients with PD compared with controls [195] and correlate with cognitive decline [212]. No differences in plasma have been found between AD and controls [49, 331].

In summary, although fluid-based α-synuclein has promise as a diagnostic and prognostic biomarker for PD and CJD, studies in AD have been relatively limited and its potential role as a biomarker is unknown (Table 1). Nevertheless, α-synuclein may prove to be useful for identifying LB pathology among AD patients, therefore, could support subject/patient selection (Table 2).

## TDP-43 pathology

TDP-43 binds both DNA and RNA and is involved in transcription and splicing. Under pathophysiological conditions, TDP-43 accumulates in the cytoplasm and is hyperphosphorylated and/or ubiquitinated, and this is characteristic of the cytoplasmic inclusions observed in ALS and in many cases of FTLD [51, 265]. TDP-43 pathology is also detected in 20–50% of AD patients [7, 50, 159], and appears to be associated with greater brain atrophy, memory loss, and cognitive impairment [50, 163]. Studies suggest that TDP-43 pathology can be triggered by Aβ peptides, and that TDP-43 contributes to neuroinflammation and may have a role in mitochondrial and neural dysfunction [50].

### Fluid biomarkers of TDP-43 pathology

#### TDP-43

A few studies have reported on CSF and plasma TDP-43 in ALS and FTLD [165, 187, 345], but research has been hampered by difficulties with detecting the protein (candidate antibodies have been reviewed by Goossens et al.) [111]. Furthermore, CSF TDP-43 appears to be mainly blood-derived, although it may be possible to enrich for brain-specific fractions of TDP-43 from exosomes in CSF [90].

One study reported elevated plasma TDP-43 in a greater proportion of AD patients compared with controls [97]. Another small study found that plasma levels of disease-related TDP-43 variants were increased in the pre-MCI stage in subjects who subsequently progressed to AD dementia [387].

Overall, research to date suggests that blood-based TDP-43 may have a role as an AD biomarker for subject/patient selection and prognosis and could be more useful than CSF TDP-43 (Tables 1 and 2). Commercial assays are available.

## Iron toxicity

Iron is important for normal functioning of the brain, but when present in excess it is known to cause neurodegeneration, for example in the genetic disorders classified as neurodegeneration with brain iron accumulation (NBIA) [135]. Studies have shown elevated iron in AD [55, 226] and MCI [372] brains, which is also replicated in animal models [210]. Iron is a redox-active biometal that has been shown to bind Aβ in vitro and cause its aggregation, while releasing hydrogen peroxide [147]. Intracellular iron can influence APP processing and bind to hyperphosphorylated tau and induce its aggregation [58]. In a recent magnetic resonance imaging study, brain iron measured by quantitative susceptibility mapping was shown to be moderately elevated in people with PET-confirmed Aβ, but highly predictive of cognitive decline over 6 years only in subjects with Aβ, suggesting that iron accelerates the clinical manifestation of the underlying pathology [16].

### Fluid biomarkers associated with iron metabolism

#### Ferritin

Ferritin is the major intracellular iron storage protein in the body and has an important role in brain iron homeostasis [333]. Inherited ferritinopathies are associated with motor and cognitive dysfunction [333], and ferritin levels are increased in AD brain tissue [58]. CSF levels of ferritin have been shown to be higher in *APOE* ε4 carriers than in non-carriers, but there was no difference in levels among subjects with AD or MCI and controls [15]. Increased CSF ferritin levels were associated with cognitive decline and predicted progression from MCI to AD, regardless of *APOE* genotype [15]. In a subsequent analysis, CSF ferritin was associated with cognitive decline in cognitively normal subjects, but the association was strongest in *APOE* ε4 carriers [14]. In the same cohort, high CSF ferritin was associated with accelerated depreciation of CSF Aβ42 in subjects with a high tau/Aβ42 ratio [13]. Plasma ferritin levels showed a modest correlation with CSF levels, but unlike CSF ferritin, there was no difference in plasma ferritin between *APOE* ε4 carriers and non-carriers [15]. In another study, plasma ferritin levels were elevated in cognitively normal subjects with Aβ pathology identified by PET when adjusted for covariates (age, sex, *APOE* ε4 status, and levels of C-reactive protein), although ferritin alone had a relatively minor effect compared with the base model (derived from logistic regression of the same covariates) [112].

In summary, the data are limited but a small number of studies suggest that both CSF and plasma ferritin may be useful as AD biomarkers (Table 1). CSF ferritin may have a role as a prognostic biomarker, whereas plasma ferritin could be used for subject/patient selection (screening) to help identify preclinical AD (Table 2); however, further studies by independent groups are needed to validate the initial findings. Commercial assays are available.

## Other neuronal proteins

### VILIP-1

Visinin-like protein 1 (VILIP-1, or VLP-1) is a neuronal calcium sensor protein involved in signalling pathways related to synaptic plasticity [115]. CSF VILIP-1 was identified through mouse gene array analyses as being abundantly produced in the brain [192]. It was subsequently associated with AD and found to correlate with CSF t-tau and p-tau [194], supporting its use as a neurodegeneration marker.

CSF VILIP-1 levels have been shown to be higher in patients with AD compared with controls in several studies [194, 230, 257, 347, 355], although one study found no significant difference [176]. The meta-analysis by Olsson et al. found VILIP-1 to have a moderate association with AD (data from four AD and control cohorts) with around 50% higher levels in AD than in controls [271]. AD patients had higher levels than MCI subjects in one study [257] but no difference was noted in a later study [17]. In a recent study of the ADNI cohort, baseline CSF VILIP-1 levels were higher in Aβ-positive (based on CSF Aβ42) MCI and AD subjects compared with both the Aβ-negative MCI and Aβ-negative cognitively normal groups [347]. No significant differences were found between any of the Aβ-positive subsets of the cognitively normal, MCI, and AD groups. CSF VILIP-1 levels decreased longitudinally in the AD group (mean follow-up was 4 years) but there were no significant longitudinal changes in any other group [347]. CSF VILIP-1 may be prognostic of future cognitive decline [355], rates of cognitive decline [357], rates of brain atrophy [356], and progression from MCI to AD [176]. In addition, studies suggest that CSF VILIP-1 can diagnostically differentiate AD from other dementias [17, 230, 355].

Data on plasma VILIP-1 are limited; plasma levels were found to be higher in patients with mild AD compared with controls in one study, although the difference was more significant in CSF than in plasma [355].

Overall, the data for VILIP-1 suggest a possible role in subject/patient selection and prognosis (Tables 1 and 2) but study results have varied so further research is warranted. Commercial assays are available.

### NF-L

Neurofilaments are intermediate filaments expressed in neurons and are particularly abundant in axons [398]. They are composed of four subunits—neurofilament light (NF-L), neurofilament middle (NF-M), neurofilament heavy (NF-H), and α-internexin in the CNS, and NF-L, NF-M, NF-H, and peripherin in the peripheral nervous system [398]. Neurofilaments are essential for the radial growth of axons during development, structural support, and the transmission of electrical impulses [398]. Recent research suggests that they are also important for normal synaptic function [399]. Abnormal aggregation and other alterations of neurofilaments are evident in several neurological diseases including AD [378, 398, 399] and in the LBs of PD [316].

CSF levels of the NF-L subunit are known to be increased in several neurodegenerative diseases, supporting its role as a marker of axonal injury [231, 286, 334]. CSF NF-L levels have been shown to be higher in AD [4, 220, 288, 334, 403] and MCI patients [403] compared with controls, and correlate with cognitive impairment and short survival time in patients with dementia [335]. The meta-analysis by Olsson et al. found CSF NF-L to have a large effect size for differentiating between AD patients and controls (data from nine AD cohorts and eight control cohorts) [271]. CSF NF-L correlates with brain atrophy [282, 403], but appears not to be specific for AD since levels are elevated in other neurodegenerative diseases, likely reflecting non-specific axonal injury [28, 103, 282]. In multiple sclerosis (MS), CSF NF-L has been shown to correlate with clinical and radiological outcomes, making it potentially useful for monitoring response to therapy [182, 249, 266, 267].

Recently, there has been great interest in the potential utility of NF-L in blood as a biomarker for several neurodegenerative diseases including AD, MS, progressive supranuclear palsy (PSP), ALS, and Huntington’s disease [43, 71, 228, 240, 302, 385, 408], as well as a marker of traumatic brain injury [224, 324]. In AD, plasma or serum levels of NF-L have been shown to be elevated compared with controls in presymptomatic individuals known to be carriers of AD-causing gene mutations [385] and subjects with MCI or AD [240, 408]. Furthermore, blood-based NF-L appears to correlate with poor cognition and brain atrophy [240, 385]. In MS, serum NF-L has demonstrated potential as a biomarker for monitoring response to DMTs and predicting relapse [71], and in PSP, plasma NF-L has been shown to predict disease progression [302].

Taken together, these findings indicate that both CSF and plasma NF-L are promising biomarkers, although the specific COU has not been determined given that changes are observed in various neurodegenerative diseases, not just AD (Tables 1 and 2). Potentially, CSF NF-L could be useful as a non-specific marker of axonal injury and for prognosis, and recent research gives hope that plasma NF-L could be used as a non-invasive biomarker for subject/patient selection (screening) and prognosis (Table 2). Commercial assays are available and IVD assays are available for CSF NF-L in Europe.

## Discussion

In addition to the established core CSF biomarkers, Aβ42, t-tau, and p-tau, several candidate fluid biomarkers show potential for clinical use in AD, particularly to support diagnosis (and clinical trial subject selection) and prognosis (or assessment of disease state) (Table 2). Of all the biomarkers reviewed, CSF Aβ42, t-tau, p-tau, and the ratio of tau/Aβ42 are already accepted for use as diagnostic biomarkers, while several other biomarkers hold promise for future use (Table 1). Further studies are needed for the validation and regulatory qualification of all these biomarkers. In addition, the relationship between the biomarkers and clinical presentation (i.e. cognitive measures), as well as the effects of patient variables (e.g. sex, *APOE* ε4 status) on biomarker changes need to be investigated.

It should be noted that only a selection of promising biomarkers has been included in this review, and many other candidates are being studied at present. As well as other protein/peptide markers and panels [206], non-protein analytes such as lipids [8], amino acids [56], and microRNAs [120, 328] are being explored. Advances in technologies such as mass spectrometry enable the precise measurement of analytes, helping to identify new candidate biomarkers [26] as well as supporting harmonization efforts for the core biomarkers [292].

Of all the possible biomarker COUs, there appears to be an unmet need for validated fluid biomarkers for drug development, especially for monitoring response to therapy and adverse reactions (Table 2). This is not surprising given the current absence of approved DMTs but highlights the need for fluid-based surrogate biomarkers of drug efficacy and safety. The important role of biomarkers in AD drug development has been highlighted in the FDA draft guidance for industry [96].

Further development of candidate biomarkers, as well as identification of new ones, would benefit greatly from a unified and coordinated approach [100, 124, 178, 269]. There is a need to reach a consensus on the areas that require the most focus and to implement effective strategies to advance the field. This effort requires collaboration among academia, industry, laboratory managers, and clinicians, at an international level.

An ever-increasing number of biomarkers are being researched, and studies have a considerable degree of heterogeneity (biomarker collection/methodology, disease diagnosis/stage of disease, and characterization of comorbid CNS diseases, especially neurodegenerative diseases), making it difficult to interpret results and establish how the biomarkers fit within the stages of AD pathogenesis. Publication bias may be a barrier in this step, as “negative” studies may be under-published. To fast-track data dissemination, a centralized database would be useful to share individual patient-level biomarker data. The Coalition Against Major Diseases (CAMD), one of 12 consortia of the Critical Path Institute (C-Path), aims to include CSF biomarker data in a central repository as part of their on-going initiative to advance regulatory drug development tools [12].

Once the data gaps are identified, studies can be designed to address the specific unmet needs. Careful planning of study design, subjects, and methodology is critical, to ensure that data gaps are appropriately addressed and that outcomes are reliable and representative of a wider population. The COU should be decided from the outset, and this will influence the subject inclusion criteria and study design. For example, studies on biomarkers for preclinical AD should enroll cognitively normal subjects with evidence of AD pathology and include longitudinal follow-ups and biomarker measurements over 5 years or more.

For studies to provide meaningful and comparable data, a concerted effort needs to be made to reduce heterogeneity in study methodologies. The development and/or update of consensus recommendations and guidelines should help in this regard, for example, by standardizing diagnostic criteria for different stages of the AD continuum, pre-analytical variables, assays, threshold values, and study designs and populations used for any given COU. The National Institute on Aging–Alzheimer’s Association (NIA–AA) is currently updating a research framework for AD, which will help to harmonize subject selection and disease staging in future studies. There have been longstanding efforts to better understand and control for pre-analytical sources of variability in CSF AD biomarkers, and consensus conferences have defined these [375]. A CSF pre-analytics consortium, sponsored by the Alzheimer’s Association is working to develop a consensus regarding remaining pre-analytical factors such as tube plastic type and other collection parameters that can be implemented into routine clinical practice. Factors that had been recognized but incompletely understood, such as the effect of tube type and CSF volume involved in transfer steps, were recently described and will help to clarify the potential impact of such factors on CSF AD biomarker measurements [386]. The International Federation of Clinical Chemistry Working Group for CSF proteins (IFCC WG-CSF) is an international joint effort to develop reference measurement procedures (RMP) and certified reference materials (CRM) with the aim of standardizing CSF biomarkers and harmonizing read-outs between assay formats [185]. To date, two Joint Committee for Traceability in Laboratory Medicine (JCTLM) -approved RMPs and three CRMs for CSF Aβ42 are available, and work on Aβ40 and tau proteins is ongoing. In parallel with the recent drive to standardize CSF pre-analytics, guidelines have also been proposed by the Biofluid Based Biomarkers Professional Interest Area (of ISTAART) for the pre-analytical processing of blood-based AD biomarkers [268]. Furthermore, “Appropriate use criteria for CSF in clinical practice” are also being developed by the Alzheimer’s Association, which will help define the use of AD CSF biomarkers by clinicians for assessment of cognitive decline and impairment.

Although biomarkers are routinely included in drug studies for understanding target engagement and for patient enrichment, the hurdles are high for biomarker adoption to inform standard of care in daily clinical practice. Health agencies have recognized the importance of biomarkers, and both the FDA and EMA have developed pathways to accelerate biomarker qualification for clinical trials [12]. To achieve biomarker qualification, evidence is needed that the biomarker can reliably support a specified manner of interpretation and application in drug development for a specifically stated COU [12]. Therefore, the aforementioned hurdles, such as data sharing and standardization of study methods, are important to address at the earliest stages of biomarker research. Once a biomarker has been shown to be useful for a specific COU in the clinical trial setting, measures can be taken to further develop it as a companion biomarker useful to practitioners.

The ultimate goal in AD is to follow the approach developed in the more advanced research field of oncology and deliver precision medicine to all patients, in such a way that diagnosis, treatment, and prevention are “tailored” to the characteristics of the individual according to the precision medicine paradigm [46, 92, 126, 127, 129, 130]. In this context, the precision medicine strategy enables a paradigm shift from the traditional “one treatment fits all” approach in drug discovery towards biomarker-guided “tailored” therapies, i.e. targeted interventions adapted to the biological profile of the individual patient. In this regard, the US Precision Medicine Initiative (PMI) (https://obamawhitehouse.archives.gov/precision-medicine) and the US All of Us Research Program (https://allofus.nih.gov/)—formerly known as and evolved from the US PMI Cohort Program (PMI–CP)—have been inaugurated recently. As is the case in most fields of medicine, important progress in detecting, treating, and preventing AD is anticipated from the implementation of a systematic precision medicine strategy. Therefore, after more than a decade of failed therapy trials and one of the lowest success rates in drug development in medicine, the time has come to launch an international Alzheimer PMI (APMI) and associate it with the US PMI and other related worldwide initiatives [92, 126, 127, 129, 130].
